# Severe Allergic Drug Reaction to Ultrasound Enhancing Agent Resulting in Cardiogenic Shock

**DOI:** 10.1016/j.jaccas.2025.106044

**Published:** 2025-11-20

**Authors:** Alexander Levy, Jason Feinman, Matthew Cagliostro, Kyle Nelson, Stamatios Lerakis, Gregory Serrao, Caroline R. Gross, Steve Liao, Lori Croft

**Affiliations:** a The Mount Sinai Fuster Heart Hospital, Icahn School of Medicine at Mount Sinai, New York, New York, USA; b Department of Anesthesiology, Icahn School of Medicine at Mount Sinai, New York, New York, USA

**Keywords:** acute heart failure, cardiac assist devices, cardiomypathy, contrast agent, echocardiography, ultrasound

## Abstract

**Background:**

Ultrasound enhancing agents (UEAs) are a commonly used tool to enhance the diagnostic quality of echocardiographic studies. Although serious allergic reactions are rare, they have been documented.

**Case Summary:**

A 39-year-old woman with NYHA functional class I heart failure developed a severe allergic reaction to UEA with decompensation into Society for Cardiovascular Angiography & Interventions stage D cardiogenic shock. Serial echocardiography demonstrated abrupt decrease in left ventricular ejection fraction from a baseline of 45% to 20%, which recovered to 35% before discharge.

**Discussion:**

Higher rates of adverse drug reactions (ADRs) are reported in observational studies of contemporary practice compared with previously published data. Cardiogenic shock as a consequence of an anaphylactic reaction to Lumason has not been previously described in the literature.

**Take-Home Messages:**

Observational data have shown that ADR to UEA has become more prevalent in the past several years, with higher rates with Lumason vs Definity. It is important to monitor patients who have ADRs to UEA and who are treated with epinephrine, especially those with underlying cardiomyopathy, as there is potential for a transition from anaphylaxis to stress cardiomyopathy and cardiogenic shock. Given the risk for significant ADR, UEA should be used selectively and only as needed.

## Visual Summary

**Figure:**
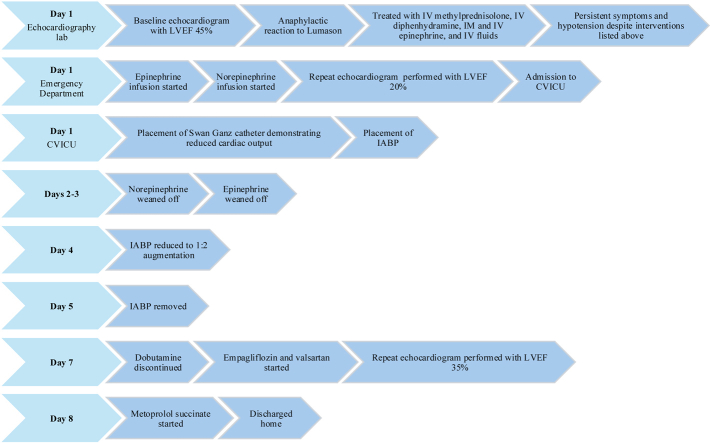

## History of Presentation: How the Patient Presented and Physical Examination

A 39-year-old woman presented for a routine outpatient transthoracic echocardiogram (TTE) because of her history of cardiomyopathy. Ultrasound images were acquired as per our laboratory’s protocol. Given the presence of breast prostheses, Lumason (sulfur hexafluoride lipid-type A microsphere; Bracco Diagnostics) ultrasound enhancing agent (UEA) was administered to improve diagnostic quality, as had been used on several of her prior TTEs. Minutes after administration of Lumason, the patient developed dyspnea and a generalized tingling sensation. Physical examination revealed an alert patient in significant distress. There was no oropharyngeal swelling or edema. No wheezing was present, and there was normal air movement on lung auscultation. Heart rhythm was regular, and there were no murmurs appreciated. There was no accessory muscle use with breathing, and although tachypneic, respirations were not labored. The skin over the neck and upper chest was diffusely erythematous. Extremities were warm, and there was no edema. The heart rate was 80 beats/min. Initial blood pressure was 76/56 mm Hg. Oxygen saturation was 98% on room air.

**Take-Home Messages**

• Observational data have shown that ADR to UEA has become more prevalent in the past several years, with higher rates with Lumason vs Definity.
• It is important to monitor patients who have ADRs to UEA and who are treated with epinephrine, especially those with underlying cardiomyopathy, as there is potential for a transition from anaphylaxis to stress cardiomyopathy and cardiogenic shock.
• Given the risk for significant ADR, UEA should be used selectively and only as needed.

With high suspicion for anaphylaxis, the patient was administered 25 mg of intravenous (IV) diphenhydramine, 125 mg of IV methylprednisolone, and 0.3 mg of intramuscular epinephrine. Intravenous normal saline was started. There was no improvement in symptoms, and while the patient remained alert and able to speak, blood pressure was undetectable. We administered 1 mg of IV epinephrine, and a subsequent blood pressure was 134/72 mm Hg. On arrival of the code team, the patient’s blood pressure was unable to be detected, and another 1 mg of IV epinephrine was administered. The patient’s blood pressure returned to 100/60 mm Hg, and she was transferred to the emergency department.

In the emergency department, an epinephrine infusion was started, and respiratory distress and erythema resolved completely. She remained hypotensive, and epinephrine was titrated up to 0.5 μg/kg/min to target normotension. Norepinephrine infusion was added and titrated up to 0.5 μg/kg/min. Despite this, her systolic blood pressure remained 80 mm Hg, and a dobutamine infusion was started, with the blood pressure stabilizing at 100/60 mm Hg.

The patient’s TTE immediately before the decompensation showed mild left ventricular (LV) dilatation with a left ventricular ejection fraction (LVEF) of 45% with global LV dysfunction, normal right ventricular (RV) size and function, and no significant valvular disease. TTE obtained upon the patient’s stabilization in the emergency department while receiving epinephrine (0.5 μg/kg/min), norepinephrine (0.5 μg/kg/min), and dobutamine (5 μg/kg/min), approximately 1 hour after the outpatient echocardiogram precipitating this decompensation, showed an LVEF of 20% with global LV dysfunction, decreased RV function, and no significant valvular disease.

After stabilization, she was then transported to a cardiovascular intensive care unit.

## Past Medical History

Past medical history is notable for NYHA functional class I heart failure with nonischemic cardiomyopathy with a baseline LVEF of 45% to 55%, a likely pathogenic variant in the *TTN* gene (c.8875C>T p.(Q2959X)); scoliosis status post-spinal fusion surgery; and 3 uneventful pregnancies, the most recent completing 6 months before her presentation for echocardiogram. Home medications include carvedilol, which she had taken the morning of presentation.

## Differential Diagnosis

Differential diagnosis of persistent hypotension included anaphylactic shock and cardiogenic shock due to stress cardiomyopathy vs acute coronary syndrome.

## Investigations

Initial laboratory results included a peripheral venous blood gas with a pH of 7.2 (normal range 7.35-7.43), a CO_2_ level of 45 mm Hg (normal range 35-45), and lactate level of 5.0 mmol/L (0.50-1.99). A complete blood count showed a white blood cell count of 25.1 × 10^3^/μL (normal range 4.5-11.0), a hemoglobin level of 14.1 g/dL (normal range 11.7-15.0), a hematocrit level of 39.8% (normal range 34.0-47.0), and a platelet count of 264 × 10^3^/μL (150-450). Serum chemistry showed a sodium level of 136 mEq/L (normal range 135-145), a potassium level of 4.1 mEq/L (normal range 3.5-5.2), a chloride level of 110 mEq/L (normal range 96-108), a HCO_3_ level of 13.1 mEq/L (normal range 22.0-30.0), a blood urea nitrogen level of 18 mg/dL (normal range 6-23), a creatinine level of 0.72 mg/dL (normal range 0.50-1.10), an aspartate aminotransferase level of 46 U/L (<35), alanine transaminase level of 30 U/L (<45), a total bilirubin level of 2.2 mg/dL (normal range 0.1-1.2), a direct bilirubin level of 0.7 mg/dL (normal range 0.0-0.8), lactate dehydrogenase level of 283 u/L (normal range 100-220), and alkaline phosphatase level of 62 U/L (normal range 38-126). The high-sensitivity troponin level was 16 ng/L (<14). This trended to a peak of 4,511 ng/L at 8 hours after the initial troponin levels. The tryptase level was 10.9 μg/L (normal range 2.2-13.2).

**Figure 1:**
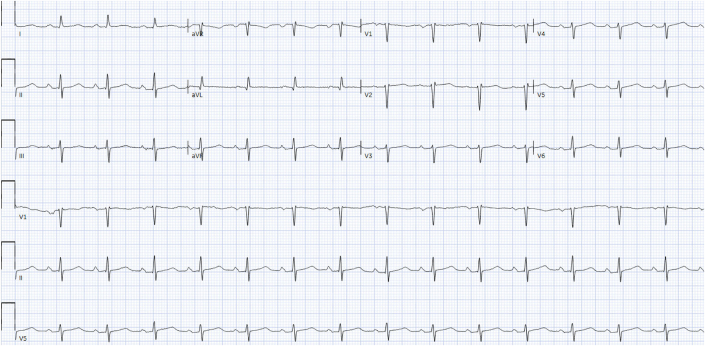
Baseline Electrocardiogram This electrocardiogram demonstrates normal sinus rhythm without acute ischemic findings.

**Figure 2:**
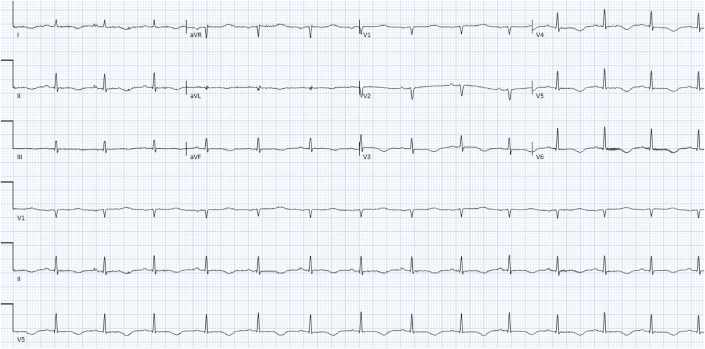
Subsequent Electrocardiogram This electrocardiogram shows development of T-wave inversions in leads I, II, aVL, aVF, and V_3_ to V_6_.

Initial electrocardiogram (ECG) demonstrated normal sinus rhythm and no acute ischemic findings, unchanged from prior (Figure 1). There were no acute ischemic findings. Subsequent ECGs over the next few hours showed development of T-wave inversions in leads I, II, aVL, aVF, and V_3_ to V_6_ (Figure 2).

## Management

On arrival to the ICU, a Swan-Ganz catheter was placed, revealing a right atrial pressure of 4 mm Hg, a pulmonary artery pressure of 26/16 mm Hg, a pulmonary capillary wedge pressure of 16 mm Hg, a mixed venous O_2_ saturation of 57.4%, a cardiac output of 2.8 L/min and a cardiac index of 1.6 L/min/m^2^ by thermodilution, and a systemic vascular resistance of 2,372 dynes/s/cm^-5^ while receiving epinephrine (0.5 μg/kg/min), norepinephrine (0.5 μg/kg/min), and dobutamine (5 μg/kg/min). Given persistently impaired cardiac output despite this support and elevated lactic acid levels, a decision was made to place an intra-aortic balloon pump (IABP).

Over the next few days, the patient was weaned off norepinephrine and epinephrine, and treatment was discontinued, guided by Swan-Ganz hemodynamics showing improving cardiac indexes. Serial echocardiograms revealed only modest recovery in the first few days with LVEF of 25% to 30%. On day 4, the IABP was reduced to 1:2 augmentation, which was well tolerated. The IABP was removed on day 5. On day 7, the patient was weaned off dobutamine, with concurrent initiation of empagliflozin and valsartan. Metoprolol succinate was added on day 8.

## Outcome and Follow-Up

She tolerated the initiation of guideline-directed medical therapy well and was discharged home on hospital day 8. An echocardiogram before discharge showed an improved LVEF of 35% and probable RV dysfunction. Follow-up is scheduled with the Advanced Heart Failure and Allergy/Immunology departments.

## Discussion

UEAs are a commonly used tool in a frequently performed diagnostic test. At our center, we performed 30,910 TTEs in 2024, using UEAs (Definity, made by Lantheus, and Lumason) in 50% of these, a higher rate than many centers. FDA approval remains only for LV opacification; however, off-label use is common and includes for evaluation of wall motion, evaluation of intracardiac thrombi, intracardiac masses, noncompaction, apical hypertrophic cardiomyopathy, complications of myocardial infarction, myocardial perfusion, and RV and LA assessments.^1^ We use UEA most frequently for LV opacification and not uncommonly for these other indications.

Life-threatening adverse drug reaction (ADR) has previously been reported to occur in <1 in 10,000 cases.^2^ The mechanism of ADR to Lumason is thought to be a type I hypersensitivity reaction. The type 1 hypersensitivity reaction mediates its effect via activation of the complement system. This type of reaction can occur even in the absence of previous exposure to the causative agent.^3^ It is not uncommon to see a normal serum tryptase level in non–IgE-mediated anaphylactoid reactions.^4^

In 2021, after a MedWatch alert of 11 cases of anaphylaxis, the American Society of Echocardiography released an Expert Consensus Statement that provided a clinical update on the mechanism of this immune response and the possible relationship to the presence of polyethylene glycol (PEG) in the UEA and did not recommend any changes to laboratory practices based on the risk-to-benefit ratio remaining extremely low.^5^

Contemporary data from clinical practices at 4 major health systems showed the rate of severe ADRs (defined as cardiopulmonary involvement) to Definity to be 1.14 per 10,000 and to Lumason to be 8.48 per 10,000. Critical ADRs (defined as involving loss of consciousness, loss of pulse, or ST-segment elevation) occurred in 0.10 per 10,000 for Definity and 3.30 per 10,000 for Lumason.^6^ The frequency of reactions with Definity remains roughly in line with historically reported rates. Given the relative safety of Definity and the value provided by UAE for LV opacification and the off-label indications highlighted by the American Society of Echocardiography, it is our practice to continue its usage as needed. However, with the higher observed rates of ADR with Lumason than with Definity, our echocardiography laboratory has made the decision to stop using Lumason. Given the increase in ADRs and ubiquitous nature of PEGylated therapeutics, understanding the mechanism of reaction and factors leading to increased incidence is of paramount importance.

We present the case of a 39-year-old patient with NYHA functional class I cardiomyopathy who experienced a Lumason-related ADR and developed Society for Cardiovascular Angiography & Interventions class D cardiogenic shock. She developed this reaction after safe administrations of Definity and Lumason in prior TTEs. Despite rapid resolution of the anaphylactic symptoms, including flushing and dyspnea, she had profound circulatory compromise. The most likely etiology of her cardiovascular decompensation is stress cardiomyopathy, supported by abrupt global LV dysfunction, diffuse T-wave inversion with lengthening QTc, and the absence of injury to other organ systems, suggesting organ perfusion was maintained throughout her resuscitation. The lack of regional ECG changes, absence of chest pain, and presence of global LV dysfunction made vasospasm or coronary dissection unlikely, and age, in addition to the aforementioned factors, made type 1 acute coronary syndrome unlikely. The clear triggering event and lack of associated symptoms made us less suspicious of myocarditis.

Stress cardiomyopathy secondary to epinephrine administration is a known phenomenon.^7^ It can occur regardless of the dose given and the route of administration. There are data to suggest that patients who are taking beta-blockers have a more severe response because of several factors, including blunted ability to compensate from a cardiac output standpoint, beta-blocker–mediated enhanced release of mast cell mediators, and blunting of the response to endogenous and exogenous epinephrine.^8^

## Conclusions

We present a case of severe allergic response to Lumason UEA resulting in stress cardiomyopathy and Society for Cardiovascular Angiography & Interventions stage D cardiogenic shock. The patient survived thanks to rapid recognition of the ADR, prompt treatment, and alertness to the transition from anaphylactic reaction to cardiogenic shock, with appropriate escalation of care and management.

**Visual Summary:**
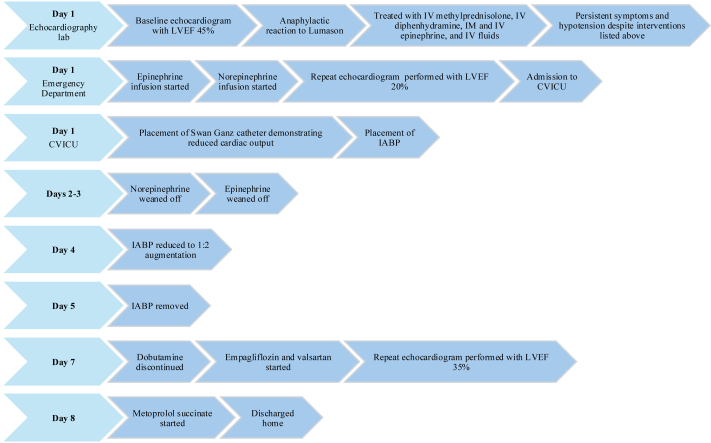
A Timeline of Events in Visual Format CVICU = cardiovascular intensive care unit; IABP = intra-aortic balloon pump; IV = intravenous; LVEF = left ventricular ejection fraction.

## Funding Support and Author Disclosures

The authors have reported that they have no relationships relevant to the contents of this paper to disclose.

## Appendix

Video 1 Baseline Transthoracic Echocardiogram

Video 2 Transthoracic Echocardiogram Performed in the Emergency Department

Video 3 Transthoracic Echocardiogram Prior to Discharge

## References

[1] Porter T.R., Mulvagh S.L., Abdelmoneim S.S. (2018). Clinical applications of ultrasonic enhancing agents in echocardiography: 2018 American Society of Echocardiography guidelines update. J Am Soc Echocardiography.

[2] Wei K., Mulvagh S.L., Carson L. (2008). The safety of definity and Optison for ultrasound image enhancement: a retrospective analysis of 78,383 administered contrast doses. J Am Soc Echocardiogr.

[3] Szebeni J. (2005). Complement activation-related pseudoallergy: a new class of drug-induced acute immune toxicity. Toxicology (Amsterdam).

[4] Mertes P.M., Alla F., Tréchot P., Auroy Y., Jougla E. (2011). Anaphylaxis during anesthesia in France: an 8-year national survey. J Allergy Clin Immunol.

[5] Lindner J.R., Belcik T., Main M.L. (2021). Expert consensus statement from the American Society of Echocardiography on hypersensitivity reactions to ultrasound enhancing agents in patients with allergy to polyethylene glycol. J Am Soc Echocardiogr.

[6] Ali M.T., Johnson M., Irwin T. (2024). Incidence of severe adverse drug reactions to ultrasound enhancement agents in a contemporary echocardiography practice. J Am Soc Echocardiogr.

[7] Madias J.E. (2016). Epinephrine administration and Takotsubo syndrome: lessons from past experiences. Int J Cardiol.

[8] Tejedor-Alonso M.A., Farias-Aquino E., Pérez-Fernández E., Grifol-Clar E., Moro-Moro M., Rosado-Ingelmo A. (2019). Relationship between anaphylaxis and use of beta-blockers and angiotensin-converting enzyme inhibitors: a systematic review and meta-analysis of observational studies. J Allergy Clin Immunol Pract.

